# Effects of combined application of benzoic acid and 1-monolaurin on growth performance, nutrient digestibility, gut microbiome and inflammatory factor levels in weaned piglets

**DOI:** 10.1186/s40813-023-00339-5

**Published:** 2023-10-19

**Authors:** Kai Wei, Xia Yang, Huasheng Zhao, Huanchun Chen, Weicheng Bei

**Affiliations:** 1 National Key Laboratory of Agricultural Microbiology, College of Veterinary Medicine, Huazhong Agricultural University, Wuhan, 430070 China; 2 ABNA Feed (Shanghai) Co.,Ltd. Zhumadian Mill, Zhumadian, Henan 463000 China

**Keywords:** Benzoic acid, Growth performance, Nutrient digestibility, Weaned piglets, 1-monolaurin

## Abstract

**Background:**

Our previous study observed that benzoic acid and 1-monolaurin have a synergistic bactericidal effect. Moreover, their improvement effect of benzoic acid and 1-monolaurin on the growth performance and diarrhea of weaned piglets was better than the two feedings alone. However, it is not clear how the combination of benzoic acid and 1-monolaurin affects the growth performance of weaned piglets. Therefore, 100 weaned piglets (mean weight 7.03 ± 1.04 kg, mean weaning age 26 d) were randomly divided into two groups: (1) basal diet control (CON); (2) basal diet supplemented with 0.6% benzoic acid and 0.1% 1-monolaurin (CA). The experiment lasted 28 days after weaning. The effects of benzoic acid and 1-monolaurin supplementation on growth performance, apparent nutrient digestibility, intestinal flora composition and function, and inflammatory factor levels of weaned piglets were investigated.

**Results:**

The feed conversion efficiency of piglets in the CA group between 15 and 28 d and 1 and 28 d after weaning was significantly higher than that in the CON group (*P* < 0.05). Additionally, the diarrhea proportion and frequency of piglets in the CA group 1–14 days post-weaning were significantly decreased (*P* < 0.05). The apparent digestibility of dry matter, organic matter and crude protein of piglets in the CA group was significantly higher than the CON group on days 14 and 28 (*P* < 0.05). The microbial composition in the cecal digesta of piglets was detected. The results indicated that the CA group piglets were significantly supplemented with *g_YRC22* at day 14 and *g_Treponema*, *g_Pseudomonas*, and *g_Lachnobacterium* at day 28 (*P* < 0.05; log LDA > 2). No significant difference was observed between the CON and CA groups in the content of short-chain fatty acids. In addition, serum IL−1β level significantly decreased at day 28 in the CA group compared with the CON group, while serum endotoxin content was significantly reduced at day 14.

**Conclusion:**

Therefore, dietary supplementation of 0.6% benzoic acid and 0.1% 1-monolaurin enhanced growth performance and nutrient digestibility, affected gut microflora composition, and decreased systemic inflammatory response and intestinal permeability of weaned piglets. These outcomes provide a theoretical basis for applying of benzoic acid and 1-monolaurin over weaned piglets.

## Background

The immune system development in piglets is immature at weaning, and stress factors, including mother-infant separation, changes in a housing environment, and diet, all affect the intestinal health of piglets. This results in digestive and absorptive capacity damage within the intestine, leading to diarrhea and decreased growth rate [1–3]. Organic acids and monoglycerides are crucial potential feed additives for pig production due to their vital molecular properties and multiple functions, such as inhibiting viral and bacterial pathogens. Among them, benzoic acid is the simplest aromatic carboxylic acid, possessing broad-spectrum antimicrobial properties [4]. Adding 0.5% benzoic acid to the diet of weaned piglets enhanced beneficial microorganisms (e.g., *Lactobacillus, Bifidobacterium*) and/or decreased the harmful ones (e.g., *Escherichia coli*) in the intestinal tract [5, 6]. When 1% benzoic acid was added to the diet, the average daily gain (ADG) of piglets was significantly increased by 15%. Moreover, the total aerobic, anaerobic, and lactic acid bacteria in the stomach were reduced. Additionally, the gram-negative bacteria and acetic acid concentration in the duodenum were reduced, along with total aerobic bacteria reduction in the ileum [7]. When benzoic acid was added to the diet at 2% or 5%, both increased the ADG, reduced the feed conversion ratio (F/G), and improved the intestinal structure and barrier function of weaned piglets [8]. Thus, benzoic acid has potent antimicrobial activity, influencing piglet growth performance by regulating gut microbial composition, structure, and function. Recent studies have revealed that combining benzoic acid with nutrients such as probiotics or plant extracts reduces diarrhea rates and enhances growth performance in weaned piglets more than benzoic acid alone [9–11]. Glycerol monolaurate (GML) is a natural, broad-spectrum antimicrobial agent with antimicrobial activity against fungi, Gram-positive bacteria, and selected Gram-negative bacteria, and is used commercially in numerous products and foods [12, 13]. The lipophilic and hydrophilic properties of 1-monolaurine help penetrate into bacteria cell walls, enabling benzoic acid entry to exert antibacterial function. Our preliminary study observed that benzoic acid and 1-monolaurin showed significant synergistic antimicrobial effects against *Escherichia coli* and *Staphylococcus aureus*. Moreover, the combined benzoic acid and 1-monolaurin concentrations were lower than those alone. However, the effects of 1-monolaurin on the growth performance and intestinal health of weaned piglets remain unreported. Thus, it remains unclear whether combining lower benzoic acid and 1-monolaurin concentrations could improve the growth performance of weaned piglets.

Depending on the results form previous experiments, the current study attempted to investigate the combined effects of benzoic acid and 1-monolaurin as feed additives on growth performance, nutrient digestibility, gut microbiome, and inflammatory factor levels of piglets. The study results would help clarify the combinatorial value of using benzoic acid and 1-monolaurin within weaning piglet diets.

## Materials and methods

### Animals, housing and diet

The study was conducted from June 2022 to August 2022 in a large-scale farm in Hubei Province, China. A total of 100 weaned piglets (mean weight 7.03 ± 1.04 kg, mean weaning age 26 d) were randomly divided into two treatment groups on the day of weaning, with 5 replicates in each group and 10 piglets in each replicate. The diets of the piglets in the two treatment groups are shown in Table 1, which were used to feed the experimental piglets from 1 to 14 days and 15 to 28 days after weaning. The control group diet was the basal diet (control, CON), and the experimental group diet was supplemented with 0.6% benzoic acid and 0.1% 1-monolaurine based on the basal diet (complex acid, CA). The experiment lasted 28 days. During the experiment, the piglets were housed in semi-enclosed nurseries with cement floors and good ventilation and warming measures, and the pen size was 10 m^2^ per pen, with 10 piglets in each pen. The piglets were fed small amounts of pellets several times a day starting at 6:00 a.m. The amount of feed remaining in the trough was recorded at 5:00 p.m., and the trough was cleaned approximately 30 min after the last meal was fed at 8:00 p.m. The piglets drank water freely. Piglets were vaccinated against Mycoplasma and African swine fever at 35 days of age. All other feeding management procedures were uniformly implemented according to the farm regulations.

**Table 1:** Ingredients and nutrient composition of diet

Ingredients	1-14d	15-28d
Maize, %	28.57	47.93
Maize Extruded, %	25	15
Low protein whey powder, %	10	4
Soybean Meal Fermented, %	10.8	5.5
Soybean Meal Dehulled, %	4	12
Soybean Extruded, %	5	5
Peruvian Fish Meal, %	5	2
Cane Sugar, %	5	2
Homogenizing Oil Powder, %	2	1
Soya Oil, %	0.5	0.5
Calcium Formate, %	0.20	0.69
Monocalcium Phosphate, %	0.67	1.31
Salt, %	0.4	0.4
Sodium Glutamate, %	0.15	0.15
Choline Chloride 50%, %	0.1	0.08
98% L-Lysine HCL, %	0.59	0.57
DL-Methionine, %	0.3	0.3
L-Threonine, %	0.35	0.29
L-Tryptophan, %	0.1	0.09
Isoleucine 90%, %	0.051	0.024
Valine 98%, %	0.197	0.158
BHT 60%, %	0.02	0.02
Premix*, %	1	1
Total, %	100.00	100.00
Calculated nutrients		
Crude Protein, %	18.50	17.98
Sodium, mg/kg	0.31	0.22
Calcium, mg/kg	0.60	0.65
Total P, mg/kg	0.60	0.65
Digestible P, mg/kg	0.44	0.46
Copper, mg/kg	121	121
Zinc, mg/kg	110	110
Digestive Energy, MJ/kg	15.06	14.61
Net Energy, MJ/kg	10.74	10.53
Digestible Lys, %	1.35	1.25
Digestible Met, %	0.58	0.55
Digestible Met + Cys, %	0.81	0.79
Digestible Thr, %	0.92	0.83
Digestible Trp, %	0.28	0.26
Digestible Ile, %	0.72	0.66
Digestible Val, %	0.93	0.86

^*^Premix provided the following per kg of diets: Vitamin A, 11972 IU; Vitamin D3, 2535IU; Vitamin E, 200IU; Vitamin K3, 3.4mg; Vitamin B1, 2.4mg; Vitamin B2, 6.70mg; Vitamin B6, 3.80mg; Vitamin B12, 0.0225mg; Niacin, 40mg; Pantothenic, 13.30mg; Folic acid, 1.06mg; Biotin, 0.122mg; Fe, 150mg; Cu, 115mg; Mn, 36mg; Zn, 85mg; I, 1.57mg; Se, 0.3mg; Sweetener, 200g; Flavor, 750g; Phytase, 5000FTU.

### Sample collection

#### Serum sample

Five medium-weight piglets were randomly selected from each group for labeling and blood collection at 6:00 a.m. on days 1 (Con group: 6.78 kg, CA group: 7.13 kg), 15 (Con group: 10.31 kg, CA group: 10.56 kg), and 29 (Con group: 15.41 kg, CA group: 17.21 kg) of the experimental period while the piglets were fasted. Twenty milliliters of blood was collected at the anterior vena cava, placed in a non-heparinized vacuum tube, centrifuged at 3000 × g for 10 min at 4 °C, and the upper serum sample was collected and stored at -20 °C for further analysis.

#### Sample of cecal contents

On the mornings of days 1, 15, and 29, piglets were fed after blood sampling. Piglets were euthanized by intravenous injection of 2 mg/kg bodyweight of chlorpromazine hydrochloride (Shanghai Hefeng Pharmaceutical Co., Ltd., Shanghai, China) approximately 30 min after feeding. According to a previously published method [14], the whole intestine was removed, and the cecal contents samples of 5 piglets in each group were collected and placed in a sterile freezable tube and immediately frozen in liquid nitrogen for the analysis of microbial quantity and microbial metabolites.

#### Diet samples and fecal samples

Representative diet samples of about 2.0 kg were taken from each stage of each treatment group. In addition, representative faeces samples were collected to determine apparent total digestive digestibility of nutrients, according to the method described by Silva et al. (2020) [11]. Briefly, fresh feces were collected from 1 piglet randomly selected from each replicate pen in the CON and CA groups by rectal palpation (also known as rectal swab collection) on days 12 to 14 and 26 to 28. After removing contaminants such as pig hair, the feces was immediately frozen at−20 °C. Fecal samples collected over three days from piglets in the same replicate pen were pooled, and a representative sample of approximately 400 g was collected and dried at 65 °C for 72 h. Fecal and dietary samples were thoroughly ground and passed through a 40-mesh sieve for apparent nutrient digestibility analysis.

### Determination of growth performance and diarrhea rate

On the mornings of days 1, 15, and 29, piglets were weighed by head, and the average BW of piglets on day 1, day 14, and day 28 after weaning was counted, and the ADG on days 1–14, days 15–28, and days 1–28 was calculated in the CON and CA groups. The piglets were fed several times a day, the total amount of feed was recorded according to the field, and the remaining amount of feed in the feed tank was recorded at 17:00 every day. The ADFI and FCR (average daily feed intake/average daily gain) of piglets from 1 to 14 days, from 15 to 28 days, and from 1 to 28 days were calculated.

During the experiment, the fecal situation of piglets was observed every day, and the anus of piglets was examined one by one to observe whether there was fecal contamination and redness. For fecal score, refer to the scoring criteria of Xiang et al. (2020): normal feces were scored as 0, pasty feces were scored as 1, semi-liquid feces were scored as 2, and liquid feces were scored as 3. If the fecal score is ≥ 2, it is considered diarrhea [15]. The proportion of diarrhea and diarrhea frequency of each group was calculated. Proportion of diarrhea (%) = number of diarrhea piglets/number of experimental piglets ×100; Diarrhea frequency (%) = (number of diarrhea piglets × number of diarrhea days)/total number of experimental piglets × number of experimental days ×100.

### Apparent digestibility of nutrient

Fecal and experimental diet samples were analyzed for dry matter (DM, method 930.15), ash (method 942.05), crude protein (CP, method 990.03), and ether extract (EE, method 996.01) according to the procedure of the Association of Official Analytical Chemists (AOAC, 2007). The organic matter (OM) content was calculated by subtracting the ash content from the DM content. Apparent digestibility of DM, OM, CP, and EE was then calculated using acid insoluble ash (AIA) content in fecal and dietary samples as endogenous indicators [16, 17], and AIA concentrations were determined according to the procedure described by Prawirodigdo et al. (2021) [17]. The formula was: Apparent digestibility of nutrient (%) = [1- (AIA_diet_ × Nutrient_feces_) / (AIA_feces_ × Nutrient_diet_) ×100, in 77which AIA_diet_ was the AIA concentration in the experimental diet, Nutrient_feces_ was the nutrient concentration in feces, AIA_feces_ was the AIA concentration in feces, and Nutrient_diet_ was the nutrient concentration in the experimental diet.

### Microbial composition in cecal digesta was analyzed by 16 S rRNA sequencing

Total genomic DNA was extracted from each cecal digesta sample using the QIAamp Fast DNA stool Minikit (Qiagen, Germany) according to the manufacturer’s instructions. Forward primer F (5’-ACTCCTACGGGAGGCAGCA−3’) and reverse primer R (5’-GGACTACHVGGGTWTCTAAT−3’) were used to amplify the V3-V4 hypervariable region of a 16 S rRNA gene. PCR amplicons were purified and quantified, and PCR products were used for library construction and then paired-end sequenced on a MiSeq platform (Illumina, United States) at Shanghai Penosen Biotechnology Co., LTD (Shanghai, China). The sequencing data were processed using QIIME 2 (version 2019.4), and the alpha diversity values of each sample were evaluated based on the observed OTUs, Chao1, and Shannon index. Beta diversity measures dependent on the weighted UniFrac distance were calculated using mothur. LDA Effect Size (LEfSe) was performed to identify bacterial taxa differentially represented between different groups at the genus or higher taxonomic level [18].

### Examination of short-chain fatty acid content in cecal digesta

Short-chain fatty acid (SCFA) concentrations in piglet cecal digesta were analyzed by a gas chromatographic method. Specifically, approximately 20 mg of cecal digesta was first homogenized in 1.0 ml of 0.5% phosphoric acid solution, and the entire sample was centrifuged at 12,000×g for 10 min at 4 °C. The 100 µL supernatant was transferred to the corresponding 1.5 mL centrifuge tube, 500 µL of MTBE solvent was added with an internal target, and swirled for 3 min. After ultrasonication in an ice bath for 5 min, centrifuge at 12,000×g at 4 °C for 10 min. The sample was injected into a gas chromatography-triple quadrupole mass spectrometer (GC-MS/MS, 8890−7000D, Agilent, USA) equipped with a DB-FFAP column (30 m × 0.25 mm × 0.25 μm). The GC-MS/MS detection parameters are shown in Table 2, and the levels of acetic acid, butyric acid, and valeric acid in cecal chymus were determined.

**Table 2:** GC-MS/MS detection parameters

Items	Parameters
Injection volume	1 µL
Front Inlet Mode	5:1
Carrier Gas	Helium
Column	DB-FFAP (30m x 0.25mm x 0.25µm)
Column Flow	1.2 mL/min
Oven Temperature Ramp	held at 50°C for 1 min, raised to 220°C at a rate of 18°C/min and kept for 5 min.
Front Injection Temperature	250°C
Transfer Line Temperature	230°C
Ion Source Temperature	230°C
Quad Temperature	150°C

### Detection of intestinal permeability markers and inflammatory factors in serum

Serum diamine oxidase (DAO) activity, endotoxin (ET), immunoglobulin A (IgA), interleukin−1β (IL−1β), interleukin−6 (IL−6), interleukin−8 (IL−8), and tumor necrosis factor α (TNF-α) were measured by ELISA. The assay kit was purchased from Nanjing Jiancheng Bioengineering Institute (Nanjing, China), and the assay method was performed strictly according to the kit instructions.

### Statistical analyses

The data of growth performance indicators, apparent digestibility of nutrients, SCFA content of cecal digesta and serum biochemical indicators were recorded and processed by Excel. Then SPSS 27.0 software was used to perform one-way ANOVA and Duncan’s multiple comparison. Chi-squared test was used to compare the diarrhea rate indicators between groups. *P* < 0.05 indicated a significant difference between groups, and *P* < 0.01 indicated a very significant difference between groups.

## Results

### Growth performance and diarrhea of piglets

**Fig. 1:**
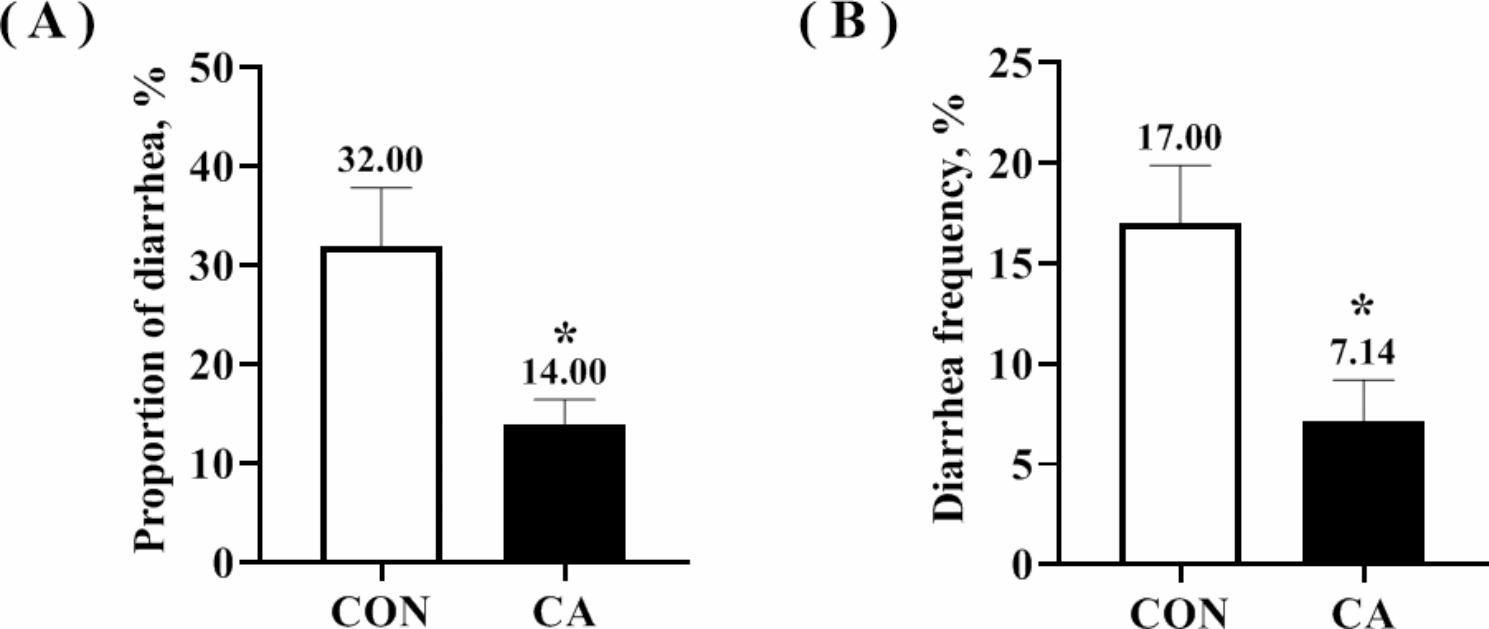
Diarrhea in piglets 1–14 days after weaning. Diarrhea proportion (**A**) and frequency (**B**) of weaned piglets. Values were expressed as mean ± SEM. *indicates significant difference between CA group and CON group, *P* < 0.05

Table 3 indicates no significant differences in BW, ADFI, and ADG of piglets in the CON and CA groups during 1−14d, 15−28d, and 1−28d after weaning (*P* > 0.05). However, the F/G of piglets in the CA group was significantly lower than the CON group (*P* < 0.05) during 15−28d and 1−28d after weaning. Comparing the piglet diarrhea in both groups, the diarrhea proportion and frequency in the CA group were significantly reduced (*P* < 0.05, Fig. 1).

**Table 3:** Growth performance of piglets

Items	Con	CA	SEM	*P*-value
BW, kg				
1d	7.05	7.03	0.19	0.97
14d	11.05	11.26	0.42	0.82
28d	16.63	17.17	0.75	0.75
ADFI, g/d				
1-14d	372.21	384.07	23.67	0.83
15-28d	596.37	590.17	37.18	0.94
1-28d	484.29	487.13	28.77	0.97
ADG, g/d				
1-14d	285.40	301.80	17.51	0.68
15-28d	398.60	422.20	24.99	0.68
1-28d	342.00	362.00	20.18	0.67
F/G, g/g				
1-14d	1.30	1.27	0.01	0.10
15-28d	1.50^a^	1.39^b^	0.02	0.02
1-28d	1.42^a^	1.34^b^	0.02	0.02

^1^BW: body weight; ADFI: average daily feed intake; ADG: average daily gain; F/G: feed conversion ratio, feed/gain;

^2^SEM: standard error of the mean;

^3 a,b^ indicate significant differences between groups, *P* < 0.05

### Apparent nutrient digestibility of piglets

**Fig. 2:**
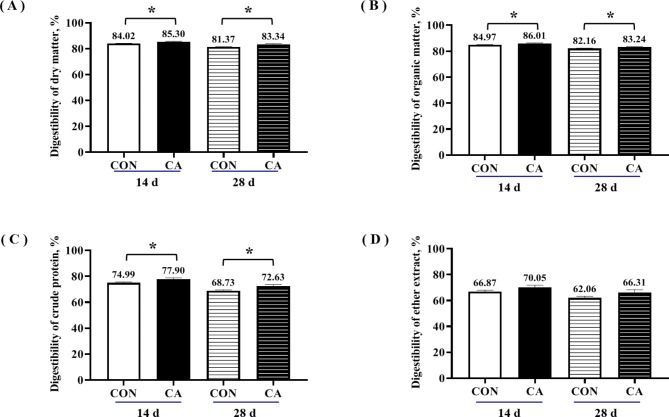
Apparent nutrient digestibility of piglets at 14 d and 28 d of the experiment. Apparent digestibility of dry matter (**A**), organic matter (**B**), crude protein (**C**), and ether extract (D) of piglets at 14 d and 28 d of the experiment. Values were expressed as mean ± SEM. *indicates significant difference between CA group and CON group, *P* < 0.05

As shown in Fig. 2, the apparent digestibility of DM, OM, and CP of piglets in the CA group was significantly higher than the CON group on the 14d and 28d when piglets were weaned on different diets (*P* < 0.05). In contrast, the EE level did not differ significantly between the groups (*P* > 0.05).

### Changes of gut microbiota in piglets

**Fig. 3:**
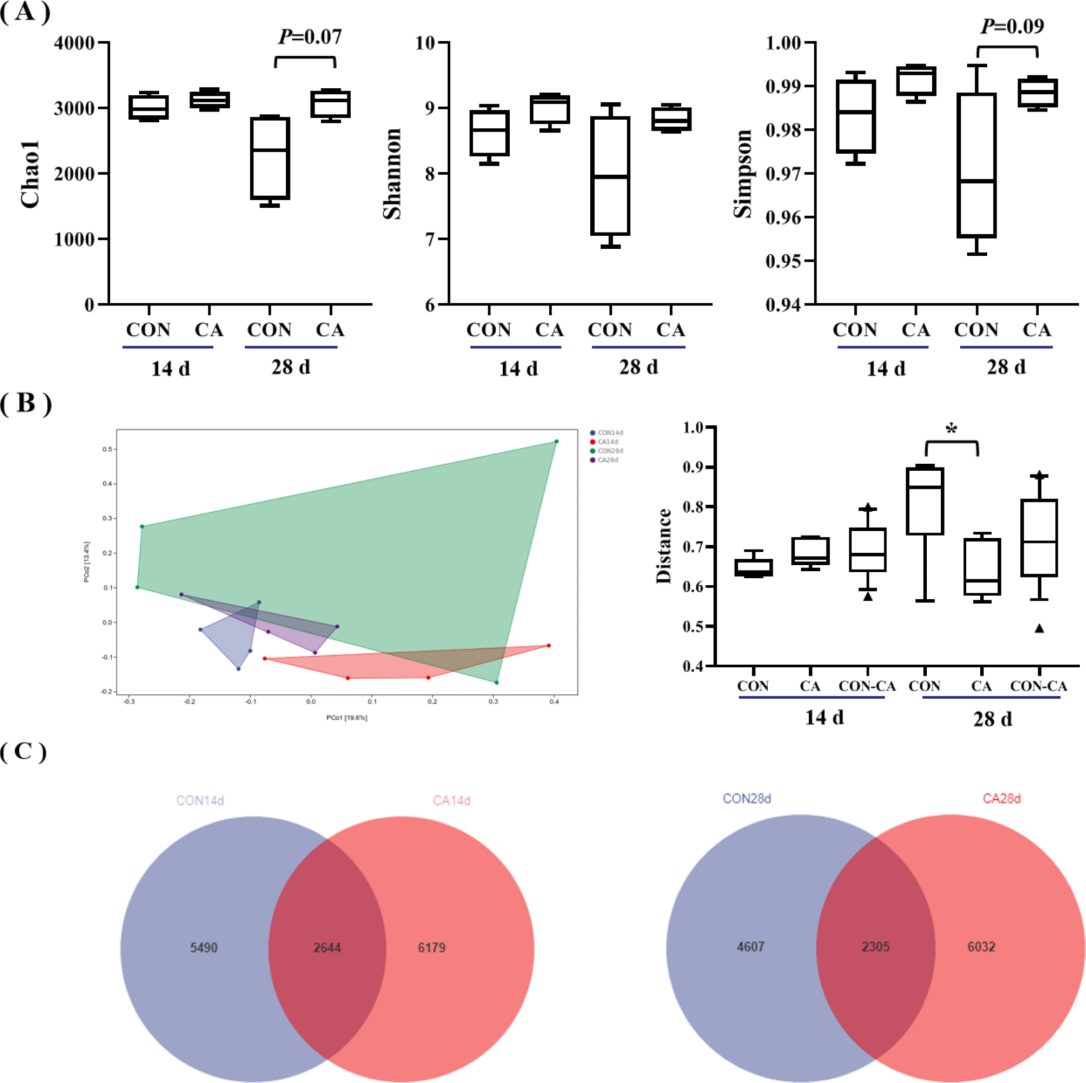
Gut microbiota composition of piglets on 14 d and 28 d of the experiment. (**A**) Boxplots of observed operational taxonomic units (OTUs) Chao 1 index, Shannon index, and Simpson indexfor the CON and CA groups piglets at 14 d and 28 d. Boxes show the medians and 10–90 percentile ranges. (**B**) The Bray-Curtis distance between samples of piglets in CON and CA group at 14 d and 28 d of the experiment was analyzed by Principal coordinate analysis.Interindividual variations were determined by average Bray-Curtis distance between individuals in the CON and CA group piglets. Intraindividual variations were determined by distance-paired CON and CA group piglets. *P < 0.05. (**C**) Wein diagram of gut microbiota composition of piglets in CON and CA groups on day 14 and 28 of the experiment

The 16 S ribosomal RNA gene sequencing analysis of the cecal digesta of piglets was performed to determine whether the growth performance improvement and apparent nutrient digestibility in the CA group are linked with gut microbiota. Around 1,659,597 high-quality sequencing reads were obtained from 20 samples, ranging between 92,381 and 119,090. Through 97% species similarity, 35,447 ASVs were obtained from piglet samples. The alpha and beta diversities were calculated. The alpha-diversity results (Chao1, Shannon and Simpson index) of the bacterial community revealed that the Chao1 index and Simpson indexes in the CA group elevated on the 28th day of the experiment (Fig. 3A). For beta diversity, the interindividual piglet variation in the CA group was lower than the CON group (*P <* 0.05, Fig. 3B) on day 28 of the experiment. Venn diagram community analysis indicated that the CA group piglets had 6179 unique ASVs compared with the CON group on 14 d of the experiment (Fig. 3C). Moreover, the CA group had 6032 unique ASVs compared with the CON group on day 28 (Fig. 3C).

**Fig. 4:**
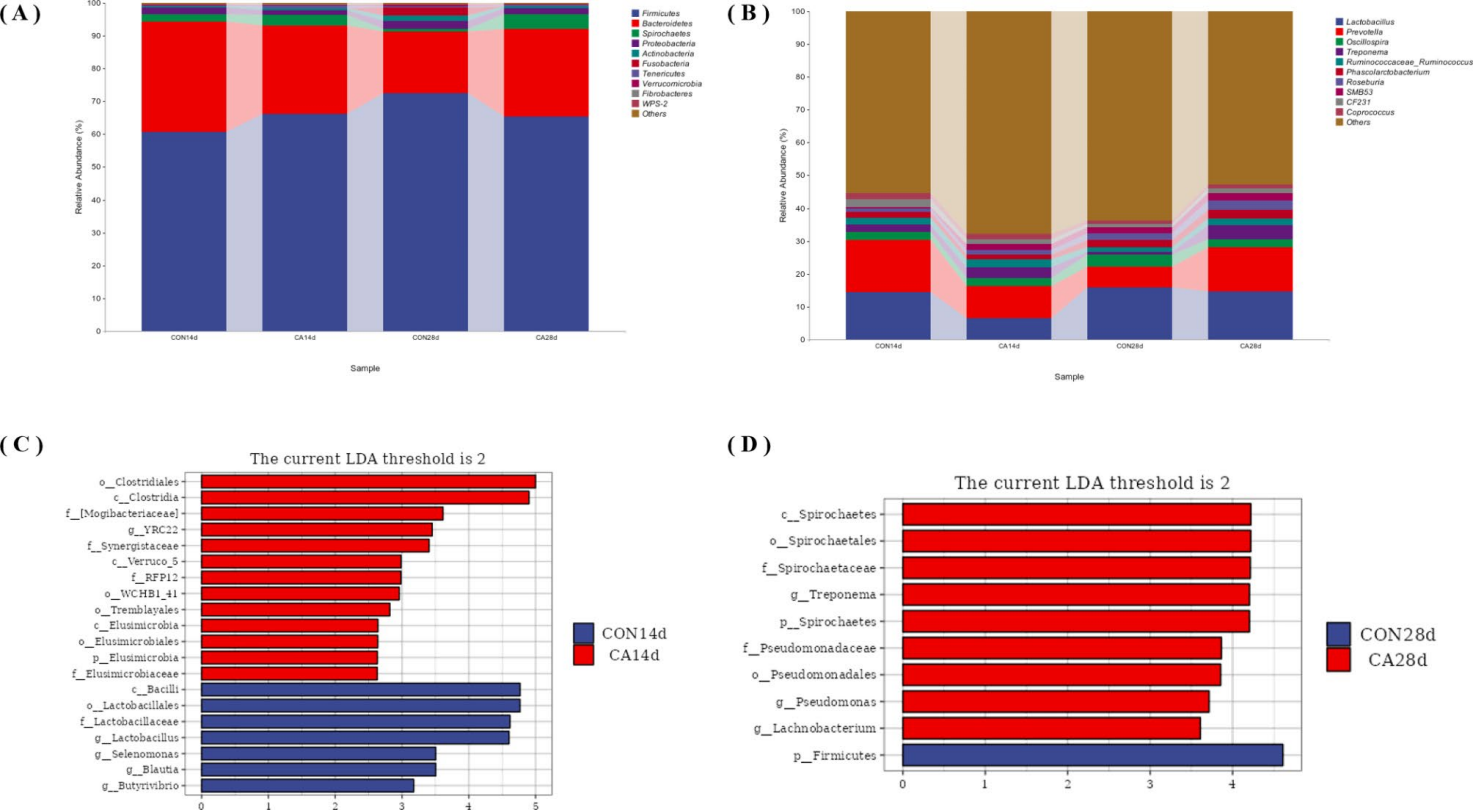
Linear discriminant analysis (LDA) score for discriminated genera in different groups of piglets at 14 d and 28 d of the experiment. Barplot of relative abundance at the phylum level (**A**) and genus level (**B**) for the CON and CA groups. The y-axis represents the relative abundance of each phylum for the two groups. LDA score for discriminated genera of piglets in the CON and CA groups on day 14 (**C**) and 28 (**D**) of the experiment. The LDA score is calculated by LDA Effect Size (LEfSe). The value suggests that it is increased in the two groups (*P* < 0.05, Wilcoxon rank-sum test, LDA > 2)

As shown in Fig. 4, the CON and CA piglet groups have different gut microflora compositions at the phylum (Fig. 4A) and the genus levels (Fig. 4B). Linear discriminant analysis (LDA) helped determine the key genera in the gut microbiota of CON and CA group piglets. *g_YRC22* was significantly enriched in the CA group on the 14th day of the experiment. *g_Treponema*, *g_Pseudomonas*, and *g_Lachnobacterium* were significantly enriched in the CA group on the 28th day (*P* < 0.05, Wilcoxon rank-sum test; log LDA > 2).

### Content of short-chain fatty acids in cecal digesta of piglets

**Fig. 5:**
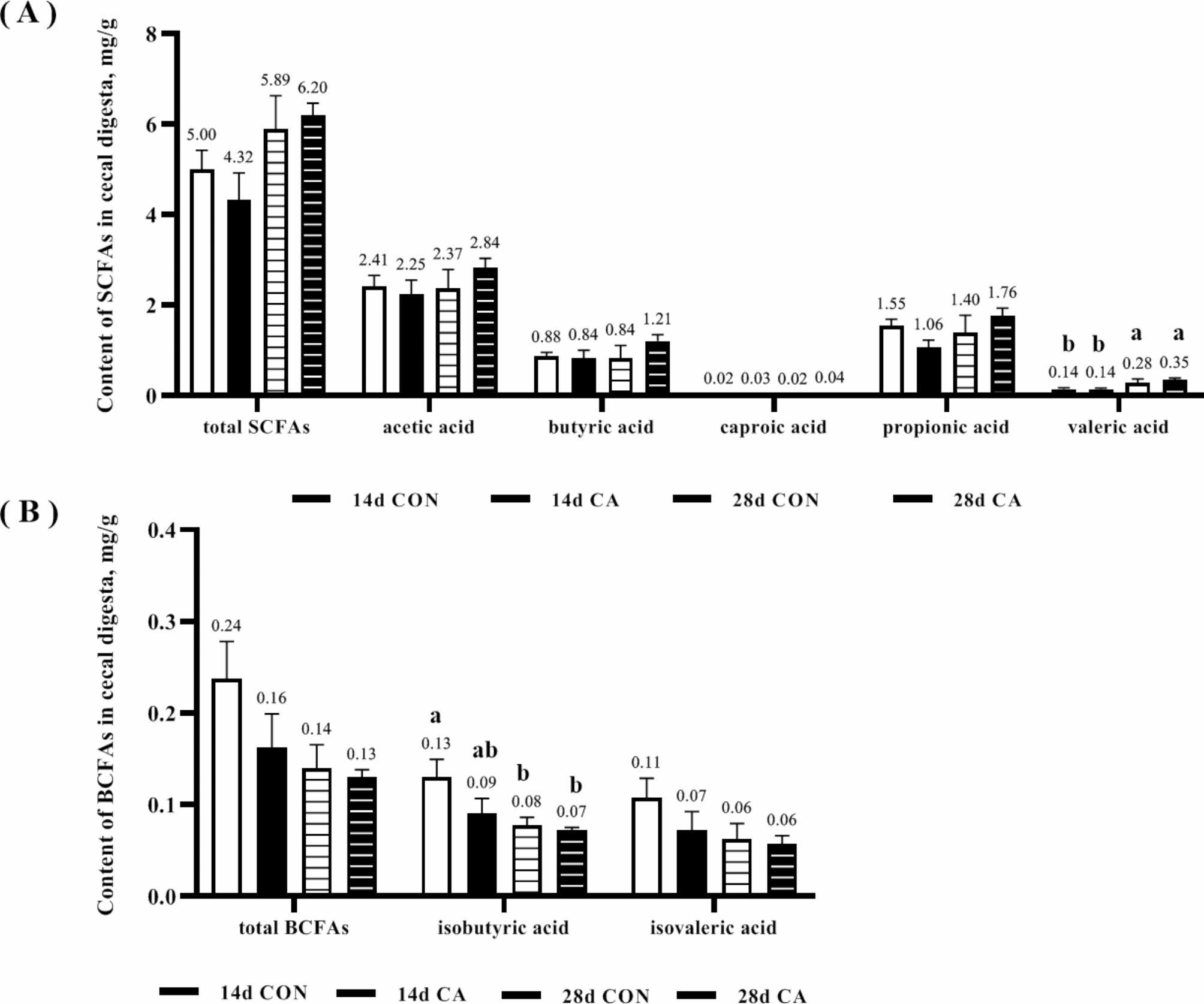
The content of SCFAs in the cecal digesta of piglets at 14 and 28 days of the experiment. (**A**) Content of SCFAs. (**B**) Content of BCFAs. Values were expressed as mean ± SEM. ^a,b^ indicates significant difference between groups, *P* < 0.05

Figure 5 showed the results of the analysis of the content of SCFAs in the cecal digesta of piglets in the CON and CA groups. Valeric acid content was significantly increased (*P* < 0.05, Fig. 5A) and isobutyric acid content was significantly decreased (*P* < 0.05, Fig. 5B) in the CON and CA groups at day 28 compared to day 14 after weaning, but there was no significant difference in the indexes between the CON and CA groups at the same time. In addition, the contents of other short-chain and branched-chain fatty acids (BCFAs) did not differ significantly between treatment groups.

### Systemic inflammation and intestinal permeability markers levels of piglets

**Fig. 6:**
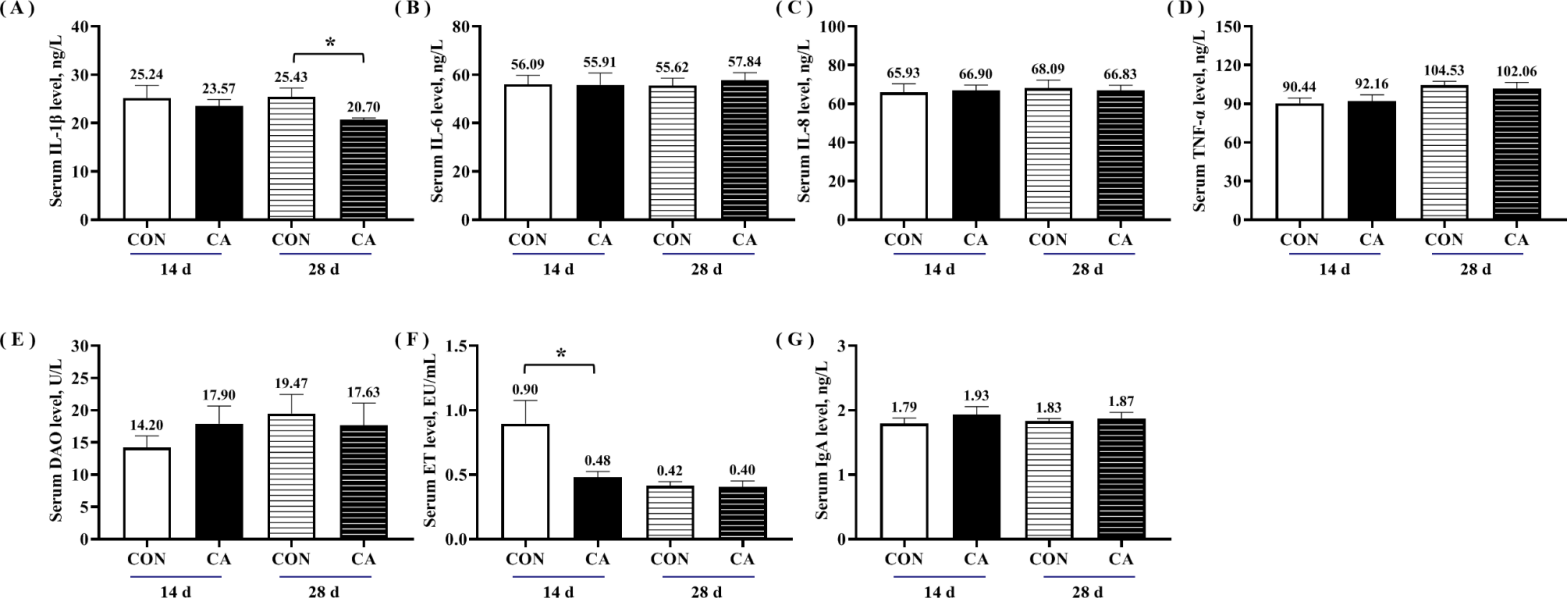
Intestinal permeability and inflammatory factor levels of piglets at 14 d and 28 d of the experiment. Serum (**A**) IL-1β, (**B**) IL-6, (**C**) IL-8, (**D**) TNF-α, (**E**) diamine oxidase (DAO), (**F**) endotoxin (ET) and (**G**) immune globulin A (IgA) level of piglets at 14 d and 28 d of the experiment. Values were expressed as mean ± SEM. *indicates significant difference between CA group and CON group, *P* < 0.05

The CA group treatment effects on systemic inflammatory marker levels among piglets are shown in Fig. 6A-D. On the 28th experimentation day, the serum levels of pro-inflammatory factor IL-1β in the CA group were significantly reduced than in the CON group (*P* < 0.05). However, diet treatment did not affect the other inflammatory factor levels (*P* > 0.05). The results of intestinal permeability markers indicated that the CA group reduced serum endotoxin (ET) content on the 14th day.

## Discussion

According to the synergistic antibacterial effect of benzoic acid and 1-monolaurin combination, their dietary supplementation effects on growth performance, nutrient digestibility, and intestinal flora of weaned piglets were investigated. The results demonstrated that the simultaneous supplementation of 0.6% benzoic acid and 0.1% 1-monolaurin elevated the feed conversion efficiency of piglets at 15–28 days and 1–28 days post-weaning. In addition, supplementation reduced the diarrhea proportion and frequency within 14 days of weaning. Moreover, our previous study observed that the combined use of benzoic acid and 1-monolaurin reduced diarrhea proportion and frequency in piglets than using the two alone (unpublished data). This finding is consistent with the results of studies in which benzoic acid was added alone or in combination with other nutrients. For example, 0.35%, 0.5%, and 1% benzoic acid supplementation can significantly enhance the ADG or reduced F/G of weaned piglets [19–21]. BW, ADG, and relative growth rate were increased by supplementng 0.75% glycerol monolaurate and oregano oil in broiler diets [22]. The reason for the ability of benzoic acid and 1-monolaurine supplementation to improve growth performance of weaned piglets may be related to its effect on nutrient digestibility and piglet diarrhea. Organic acids are an attractive alternative to enhance nutrient digestibility in the pig and poultry industry. Their multifunctional effects include lowering gastric pH, enhancing gastric retention time, stimulating pancreatic secretions, impacting mucosal morphology, and behaving as substrates in intermediate metabolism, improving digestion and absorption [23]. The dietary supplementation using benzoic acid and 1-monolaurin in our study enhanced the apparent digestibility of DM, OM, and CP in weaned piglets for 28 days post-weaning. This result was inextricably linked to the ability of benzoic acid to significantly decrease the pH of chyme in the stomach and jejunum, increase digestive enzyme levels, and increase the villus height and/or reduce the mucosal crypt depth of the duodenum, jejunum, and/or ileum of piglets [5, 24, 25]. In addition to the increased nutrient digestibility of benzoic acid, the antibacterial and anti-inflammatory effects of 1-monolaurin might be beneficial in reducing piglet diarrhea [12, 13, 26], which collectively improves the growth performance of weaned piglets.

The composition of the gut microflora in the cecum of piglets was significantly altered by the addition of benzoic acid and 1-monolaurin to the diet. On the 14th day, *g_YRC22* was significantly enriched in the CA group. *g_YRC22* is almost undetectable in the conservation and lactation stage. However, it can rapidly become a dominant bacterium and colonize the intestines of growing-finishing pigs [27]. Benzoic acid and 1-monolaurin supplementation rapidly enriched *g_YRC22* in weaned piglets within 14 days post-weaning. Therefore, benzoic acid and 1-monolaurin promoted intestinal flora stability with rapid colonization of dominant bacteria. In addition, we found a simultaneous enrichment of the conditionally pathogenic bacteria *g_Treponema* and *g_Pseudomonas* and the beneficial bacterium *g_Lachnobacterium* in the CA group on day 28 of the experiment. Unfortunately, we could not obtain more information on the *g_Treponema* and *g_Pseudomonas* that were enriched in the CA group to determine whether these two genera had harmful effects under the current data conditions. In addition, no significant difference was found in the SCFAs content of piglets in the CON and CA groups. In short, benzoic acid and 1-monolaurin supplementation stabilized the microflora fluctuation induced by weaning of piglets and promoted rapid colonization of the dominant bacteria, but had no significant effect on the content of SCFAs.

Weaning stress activates the intestinal immune system and synthesizes many pro-inflammatory cytokines, such as TNF-α, IL-1β, IL-6, and IL-8. Cytokines overproduction can cause intestinal damage and dysfunction [28]. Our study observed that dietary supplementation using benzoic acid and 1-monolaurin reduced serum IL-1β and ET levels in weaned piglets. This may correlate with 1-monolaurin’s ability to enhance antiviral activity and inhibit inflammatory factor release [29]. In vivo studies deciphered that monolaurin supplementation alone in piglets decreased intestinal pathological damage and IL-8 and TNF-α levels, improving growth performance [30]. Thus, dietary supplementation of benzoic acid and 1-monolaurin decreased systemic inflammatory response and intestinal permeability. This could be an essential reason for improving the growth performance of weaned piglets.

## Conclusion

The dietary supplementation of 0.6% benzoic acid and 0.1% 1-monolaurin improved the growth performance of weaned piglets and reduced the diarrhea rate post-weaning. This effect could be associated with increased nutrient digestibility, decreased systemic inflammation and intestinal permeability, and gut microflora stability in piglets.

## Data Availability

The datasets used and/or analyzed during the current study are available from the corresponding author on reasonable request.
